# Temozolomide encapsulated and folic acid decorated chitosan nanoparticles for lung tumor targeting: improving therapeutic efficacy both *in vitro* and *in vivo*

**DOI:** 10.18632/oncotarget.22791

**Published:** 2017-11-30

**Authors:** Kaidi Li, Naixin Liang, Huaxia Yang, Hongsheng Liu, Shanqing Li

**Affiliations:** ^1^ Department of Thoracic Surgery, Peking Union Medical College Hospital, Peking Union Medical College and Chinese Academy of Medical Sciences, Beijing 100730, China; ^2^ Department of Rheumatology, Peking Union Medical College Hospital, Peking Union Medical College and Chinese Academy of Medical Sciences, Beijing 100730, China

**Keywords:** temozolomide, chitosan, lung cancer, non-small-cell lung cancer, targeted drug delivery

## Abstract

Folic acid-conjugated temozolomide (TMZ)-loaded chitosan nanoparticles (CS-TMZ-FLA-NP) were developed to target lung cancer in the anticipation that folic acid would increase the affinity of nanoparticles for cancer cells. CS-TMZ-FLA-NP showed the highest anti-proliferative effect on the lung cancer cells in comparison to free TMZ and CS-TMZ-NP (nanoparticles without folic acid). A cellular uptake assay was performed on two different cell lines, L132 and A549. Cellular uptake efficiencies of CS-TMZ-NP and CS-TMZ-FLA-NP were found to be concentration-dependent in both cell lines. CS-TMZ-FLA-NP produced a 2.5 fold greater accumulation of TMZ than CS-TMZ-NP in both cell lines. CS-TMZ-FLA-NP maintained a significantly higher deposition of TMZ in lung tissue (approximately 7.5 μg/g of lung tissue) when compared to free TMZ and CS-TMZ-NP. Mice treated with CS-TMZ-FLA-NP had a 100% survival rate with significant suppression of tumor growth. Histopathological and immunohistochemical studies also demonstrated that CS-TMZ-FLA-NP had superior anticancer activity compared to the other two treatments. Our results indicate that CS-TMZ-FLA-NP can effectively facilitate targeting to human lung cancer cell lines *in vitro* and to lung tumors *in vivo* in a sustained manner and so improve the therapeutic efficacy of TMZ.

## INTRODUCTION

Lung cancer is a malignant lung tumor also known as pulmonary carcinoma. This carcinoma is characterized by metastasis, which is the uncontrolled growth of cells in tissues possibly spreading beyond the lungs into nearby tissues or to a variety of body parts [1]. Lung cancers can be broadly classified as small cell lung carcinoma (SCLC) and non-small-cell lung carcinoma (NSCLC). NSCLC is further classified based on the tissue type affected (epideroid, large cell, broncho-alveolar, adenocarcinoma and squamous cell carcinomas). Long-term continuous consumption of tobacco, tobacco-related products and inhalation of smoke are the major extrinsic factors that are known to cause lung cancer. In addition, genetic factors, exposure to harmful gases like radon as well as exposure to asbestos and air pollutants are some of the risk factors for the development of lung cancer [2]. The major and most common symptoms of lung cancer include coughing, shortness of breath, chest pains and weight loss. Nearly 23% of the total cancer-related deaths currently occurring globally are due to lung cancer. One survey conducted in the USA concluded that out of approximately 220,000 patients with a confirmed diagnosis of lung cancer nearly 85% of cases were NSCLC, with the remaining 15% diagnosed as SCLC. Survival rates are low in cases of lung cancer due to late diagnosis [3]. Current treatment strategies for lung cancer are generally dependent on the stage as well as the malignancy type at the time of diagnosis. They include chemotherapy, surgery or radiation therapy [4]. At the very initial stages of SCLC, chemotherapy and radiation therapy are the only options. NSCLC, however, fails to respond to these treatments and so surgery and gene therapy remain the only options. Killing of healthy cells, the requirement for very high doses to treat tumors and the difficulty in removing cancerous tissues are the main drawbacks of radiotherapy, chemotherapy and surgery for the treatment of this type of cancer, respectively [5]. Very limited access to the deeper cancerous tissues of the lungs is a major difficulty of conventional lung cancer therapies, leading to further treatment complications [6]. In view of all of these limitations as well as the mortality rates from lung cancer there is an urgent need for the development of a therapeutic approach that can increase the efficacy.

Temozolomide (TMZ) has been found to be clinically active against experimental cancer models along with pre-clinical models of NSCLC, ovarian, breast, prostate, head and neck cancers [7]. TMZ has an acceptable safety and efficacy profile that is mainly characterized by myelosuppression, which rarely requires the discontinuation of therapy [8]. One of the greatest advantages of using TMZ is that there are rarely adverse respiratory reactions. In addition to this benefit, TMZ has recently been evaluated in Phase I and Phase II clinical studies for NSCLC patients and TMZ induces sustained pro-autophagic effects in cancer cells leading to indirect apoptosis [9]. Therefore, TMZ could overcome the intrinsic resistance of a number of types of cancer, including NSCLC, glioblastomas, melanomas, and pancreatic cancer [10]. TMZ is an oral alkylating agent that is quickly hydrolyzed to active 5-(3-methyltriazen-1-yl) imidazole-4-carboxamide (MTIC) at a neutral as well as an alkaline pH. This alkylating agent produces O_6_-alkyl-guanine (O_6_-AG) lesions on DNA [11]. TMZ is completely and rapidly absorbed after oral administration with peak plasma concentrations reached within one hour. Consumption of food reduces the rate and the extent of TMZ absorption [12]. This alkylating agent has a single agent efficacy in SCLC. TMZ crosses the blood-brain barrier, which could be of significance as brain metastasis is common in this lung cancer. However, conventional drug delivery of chemotherapeutic agents is associated with undesirable side effects on healthy tissues and cells. Therefore, development of a delivery system to minimize any adverse effects is of significant importance.

Nanoparticulate drug delivery technology holds great promise for the successful enhancement of therapeutic efficacy of numerous anticancer molecules. The recent expansion of nanotechnology has provided a range of nanomaterials that are suitable for applying to lung cancer therapy [13]. The nanoparticles have ingrained tiny nano dimensions which cache in malignant cells. Nanoparticles positively alter biodistribution, thereby increasing therapeutic efficacy and reducing the nonspecific toxicity of potent anticancer drugs [14]. Nanoparticles offer ideal drug delivery technology due to their biocompatibility, ability to protect nucleic acids from degradation and ability to deliver therapeutic genes to cancerous cells *in vivo* [15]. Apart from all these advantages, polymeric nanoparticles are capable of producing a sustained release profile of drugs combined with all the features of a high loading capability of medically helpful compounds and imaging agents. This is due to the high surface area to volume ratios of nanoparticles [16].

Chitosan (CS) is a linear polysaccharide obtained by treating shrimp (and crab, lobsters, etc.) shells with alkaline sodium hydroxide. CS and its derivatives serve as a natural polymer material with excellent biodegradability, biocompatibility and nontoxic properties. Due to these properties, it has been extensively used in the medical, food, chemical, agricultural and biotechnological fields. For the same reason, in recent years CS has been explored as a nanoparticulate drug delivery carrier [17–19]. A folate receptor (FR)-mediated drug delivery system has been widely developed in the past few decades. The FR is mainly overexpressed in combination with glycosyl phosphatidylinositol; hence there is a membrane glycoprotein connection in malignant tumors and in many types of cancers, including ovarian and pulmonary cancers when compared to healthy cells [20]. Folic acid (FLA) serves as the most suited ligand due to its high affinity for the FR. FLA and folate conjugates have been proved to give significantly improved delivery to FR-positive tumor cells. FLA or its conjugates combine with FR located at the surface of cancer cells and are internalized to intracellular compartments to form endosomes [21]. It has been demonstrated that as long as the FR is not exposed to circulating FR-mediated drug, the desired specific targeting and required drug accumulation does not take place in cancer cells [22].

To improve drug delivery efficiency, in this study, we developed the folic acid-conjugated-TMZ-loaded-chitosan-nanoparticles (CS-TMZ-FLA-NP), which deliver TMZ to treat pulmonary carcinomas via inhalation. Moreover, the encapsulation efficiency (EE), particle size, cellular uptake, drug deposition and antiproliferative effect of CS-TMZ-FLA-NP on lung cancer cells both *in vitro* and *in vivo* were investigated.

## RESULTS

### Synthesis of CS-TMZ-FLA-NP

**Figure 1:**
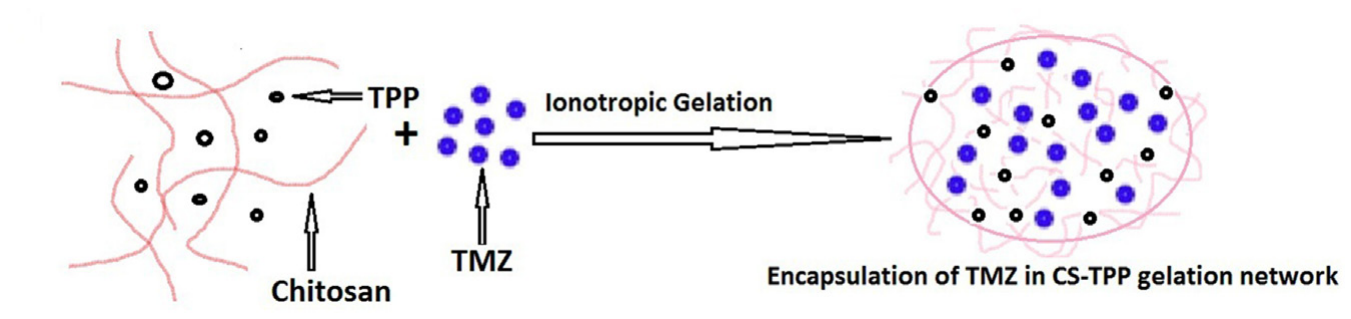
Formation and entrapment of TMZ in CS nanoparticles

**Figure 2:**
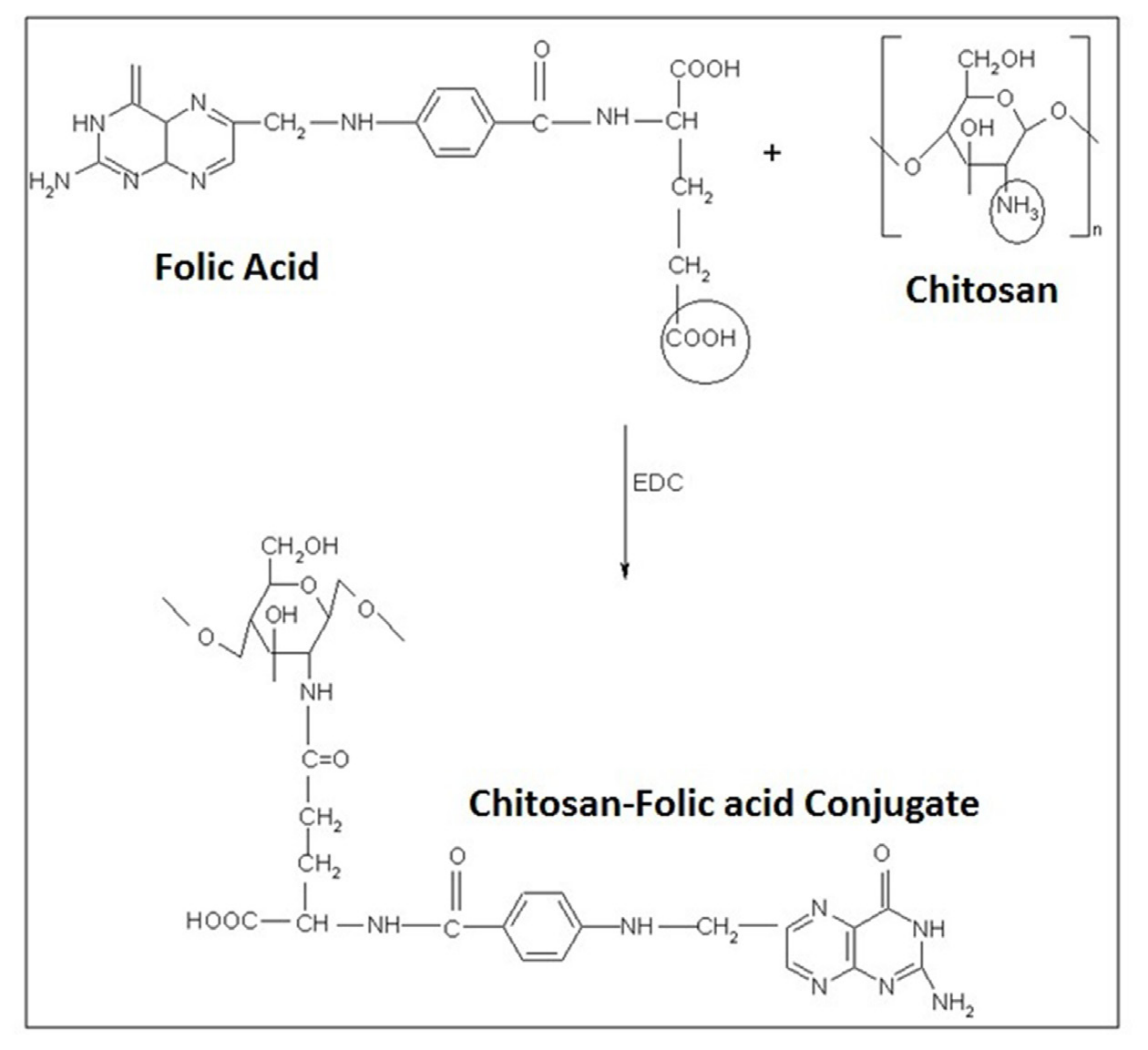
The chemistry of CS-FLA conjugate formation

**Figure 3:**
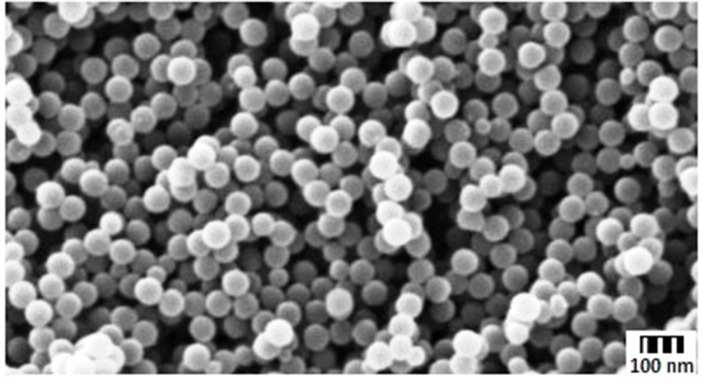
Surface morphology of nanoparticles: SEM image of CS-TMZ-FLA-NP showing that the morphology was spherical and smooth

The formation and entrapment of TMZ in CS nanoparticles are illuminated in Figure 1 and the chemistry of CS-FLA conjugate formation is displayed in Figure 2. Diameter, density and surface properties or shape determine the efficacy of nanoparticle deposition in lung tissue following inhalation. The surface of nanoparticles was analyzed by FESEM. Nanoparticles were found to be spherical in shape with smooth surfaces and distinctly separated from each other. The surface morphology of the nanoparticles in the present study is shown in Figure 3.

### Drug release *in vitro*

**Figure 4:**
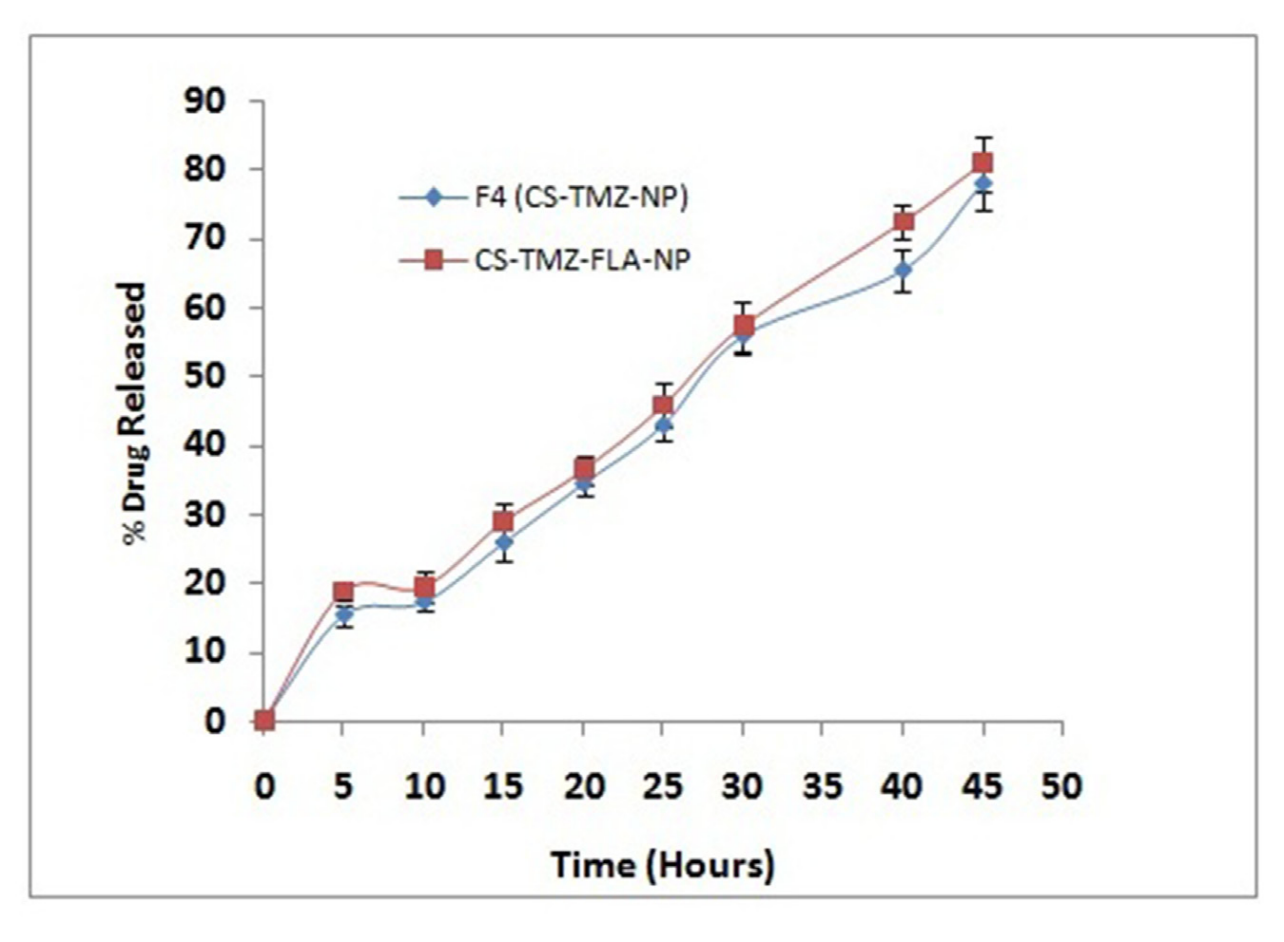
TMZ release from nanoparticles: TMZ release from optimized formulation F4 (CS-TMZ-NP) and CS-TMZ-FLA-NP in phosphate buffered saline, pH 7.4, *in vitro*, showing the sustained release of the drug No difference in the release pattern was observed between CS-TMZ-NP and CS-TMZ-FLA-NP particles, indicating that the substitution of FLA in nanoparticles did not alter the pattern of release of TMZ.

TMZ released from the optimized formulation (F4) was studied in phosphate buffered saline (pH 7.4). The optimized formulation showed an initial burst of release of 12.45% of the total from CS-TMZ-NP, followed by a slower gradual release of the drug (∼77.89%) towards the end of the 40 h study period. However, CS-TMZ-FLA-NP prepared from the optimized formulation (F4, CS-TMZ-NP) also showed a sustained drug release pattern (∼77.89%), as shown in Figure 4. Table 1 shows the comparative results with various nanoparticulate formulations. This pattern clearly indicated the tendency of sustained drug release from nanoparticles, which could be essential to cancer treatment. Drug release kinetics was studied by applying various mathematical models, including zero order, first order, the Higuchi and Koremeyer-Peppas models. The best fit model for drug release from any system can be confirmed from its correlation coefficient (R^2^); the kinetic model with the higher R^2^ is considered to be the best fit model. Out of these four models the Higuchi model (r =0.987) was found to be the best fit, indicating a diffusion-based drug release pattern. The Higuchi model is based on the hypothesis that the initial drug concentration present in any matrix system is much higher than that of dissolved drug, so drug diffusion from the matrix system can take place in only one dimension, swelling of the matrix and dissolution are negligible, drug diffusivity is always constant and sink conditions are always maintained [23]. The slope of the optimized formulation (n) was higher than 0.89 (n = 1.0562; this shows super case–II transport). In such cases drug, release takes place by both diffusion and relaxation of the polymer chain. In sustained release drug delivery systems, the drug will be released to the tumor tissue at a gradual and constant rate.

**Table 1:** Characterization of various nanoparticle formulations

Parameters	CS-TMZ-NP	CS-TMZ-FLA-NP	Blank CS-NP	Blank CS-FLA-NP
Particle size	92.50 nm	93.81 nm	89.50 nm	90.56 nm
PDI	0.112	0.145	0.085	0.095
Zeta potential	20.12 ± 4.5	19.37 ± 2.8	20.20 ± 3.5	14.7 ± 3.8
Drug release _at40hrs_	77.89 %	80.95 %		

### Statistical analysis of encapsulation efficiency (EE)

The EE of drug-loaded nanoparticles was evaluated by measuring the amount of non-entrapped drug present in the clear supernatant solution obtained after centrifugation. The range of EE was found to be from 75.32 to 97.85, as shown in Table 2. EE varied with the concentration of the independent variables. EE of TMZ in CS nanoparticles showed excellent results at the +1 level of CS concentration. In this case, the EE of the optimized formulation was found to be 97.85%. Furthermore, the EE of CS-TMZ-FLA-NP prepared from the optimized formulation did not show a significant difference in EE (∼96.78%). The polynomial equation was derived by considering the effect of the independent variables on the dependent variables.

**Table 2:** Formulation of TMZ-CS nanoparticles using a 2^3^factorial design

Batches	Factor			Response		% DL
	A	B	C	Y1	Y2	
F1	-1	-1	-1	85.93	235.54	20.12
F2	+1	-1	+1	78.80	334.56	19.20
F3	-1	+1	-1	90.92	167.34	20.50
F4	+1	+1	+1	97.85	92.50	29.50
F5	+1	+1	-1	92.87	110.45	26.90
F6	-1	+1	+1	91.67	135.76	23.45
F7	-1	-1	-1	75.32	482.50	18.75
F8	+1	-1	+1	93.26	98.78	25.57

The polynomial equation for EE (Y1) is given by:

Y1= + 49.50+17.37A+5.55B+8.48C

Where Y1, A, B and C represents EE, CS, TPP and Tween 80 concentrations, respectively. From the model with an F-value of 522.90, it is clear that the model is statistically significant. Positive and negative values in the above equation represent synergistic and antagonistic effects of the independent variables, respectively [24]. The correlation coefficient (R^2^) of Y1 was 0.9956, clearly suggesting the 2FI model, as shown in Table 3. Also, a statistically significant effect of the model can be confirmed from the p value for the Y1 model (p< 0.001). All independent variables were found to be statistically significant with A (p<0.0001), B (p=0.0022) and C (p=0.0001), as shown in Table 4.

**Table 3:** Summary of regression analysis results for responses Y1 and Y2

Models	R^2^	Adjusted R^2^	Predicted R^2^	Std. Dev	Press	Remarks
Response Y1						
Linear	0.8235	0.8421	0.8014	4.35	29.20	.......
2FI	0.9956	0.9953	0.9789	2.47	49.00	Suggested
Quadratic	0.6987	0.83.45	0.7235	2.44	25.45	
Cubic	0.8976	0.8262	0.9284	3.67	40.52	.......
Response Y2						
Linear	0.8888	0.8562	0.8590	3.87	10.00	.......
2FI	0.9730	0.9882	0.9834	1.77	20.84	Suggested
Quadratic	0.9120	0.8720	0.8250	3.67	10.00	.......
Cubic	0.8925	0.8725	0.8290	4.50	14.40	.......
Regression equations of the fitted models						
Y1= +49.50+17.37A+5.55B+8.48C						
Y2= +78.12 + 4.11 A+4.14B+4.14C						

**Table 4:** ANOVA of models for Y1 and Y2

Source	DF	Sum of squares	Mean square	F value	P value
Model for Y1	3	3777.38	1397.46	522.90	<0.0001
A	1	2002.23	2314.13	861.00	<0.0001
B	1	253.22	156.12	45.00	0.0022
C	1	513.31	600.13	125.00	0.0001
Model for Y2	3	420.74	236.58	243.23	0.0002
A	1	302.74	398.74	320.73	<0.0001
B	1	56.17	32.17	28.20	0.0057
C	1	97.83	90.83	47.75	0.0012

**Figure 5:**
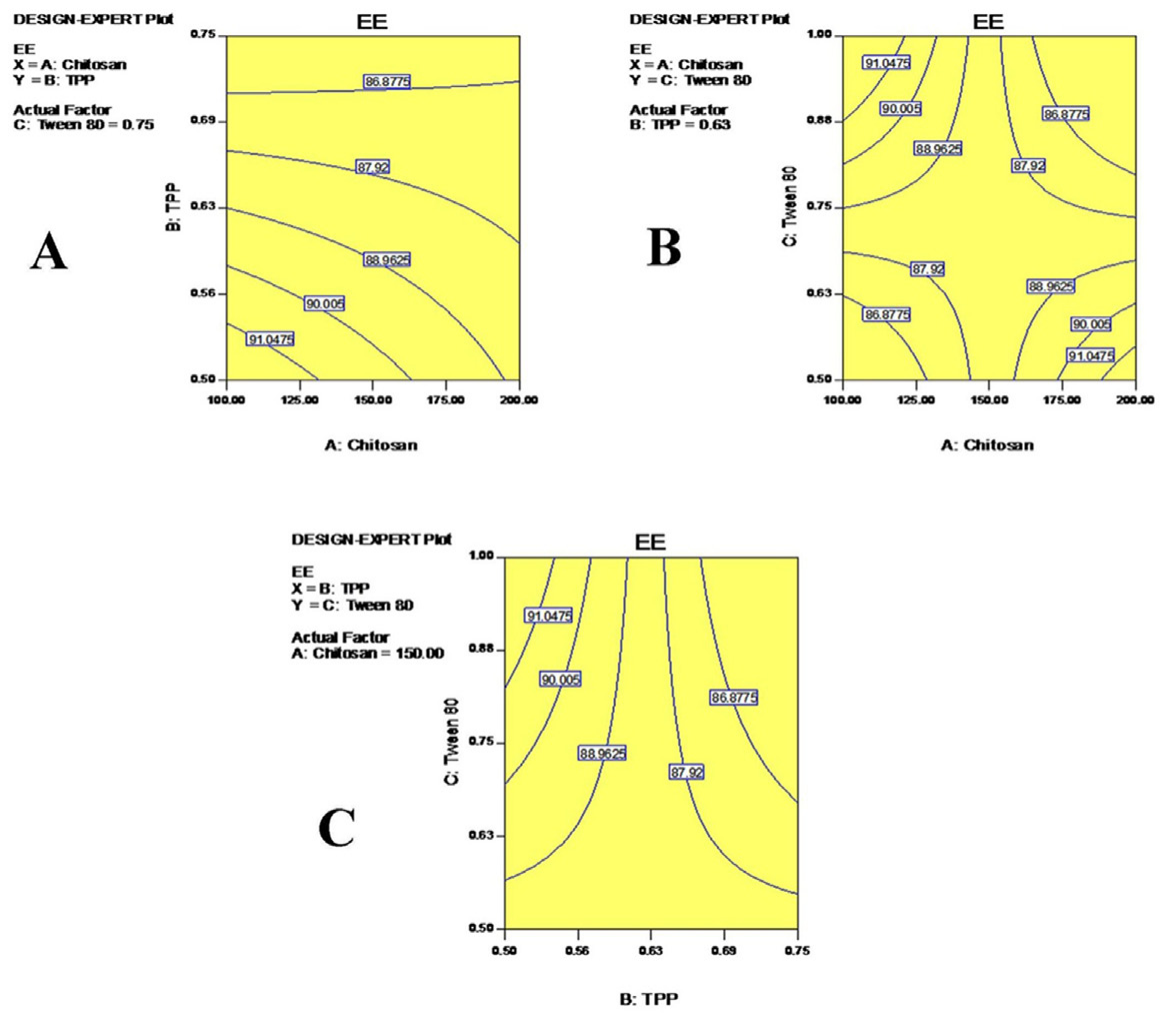
Contour plots for encapsulation efficiency (EE): Showing the effects of CS and TPP **(A)**, CS and Tween 80 **(B)** and TPP and Tween 80 **(C)** on the efficiency EE of TMZ.

The enhancement of EE was due to the ability of CS to form a gel with TPP [25]. The low EE was attributable to the lesser amounts of CS and TPP used in nanoparticle formulations. A relatively low viscosity of CS resulted, due to the lower concentration of CS leading to the leaching of TMZ from the nanoparticles that showed lower EE in such batches [26]. The enhancement of EE could be attributed to the higher concentration of the polymer present in the system with respect to the drug concentration. Higher concentrations of the polymer formed a denser mass of the drug polymer dispersion matrix, which helped to entrap the drug molecule in the nanoparticles. Nanoparticles prepared with greater amounts of cross-linking agent showed increases in EE values [27]. This could be due to an increase in the density of cross-links, which might prevent the leaching out of the drug molecule during nanoparticle formation. Contour plots are shown in Figure 5.

### Statistical analysis of particle size (PS)

An optimal size of nanoparticles is the major prerequisite for intratumoral distribution and cellular internalization. A smaller nanoparticle size increases their surface area, leading to maximum access to the tumorous region of lungs [28]. It has been reported that particles of size less than 100 nm could effectively be deposited in the alveolar region (up to 50%) leading to improvements in the chemotherapeutic activity against resistant lung cancer [29]. In the present study, a Malvern particle size analyzer (DVS) showed nanosized particles with a very narrow PDI. CS-TMZ nanoparticles were found to be in the range of 92.50 nm (F4) to 482.50 nm (F7) with PDI values from 0.120 to 0.384. TMZ loading increased the size of the nanoparticles. An inverse relationship was found between concentration of cross-linking agent (TPP) and the particle size. Even after the incorporation of FLA to nanoparticles, no significant difference in particle size was found (∼95.60 nm). Particle size (Y2) was found to be given by the polynomial equation:

Y2= +78.12 + 4.11 A+4.14B+4.14C

**Figure 6:**
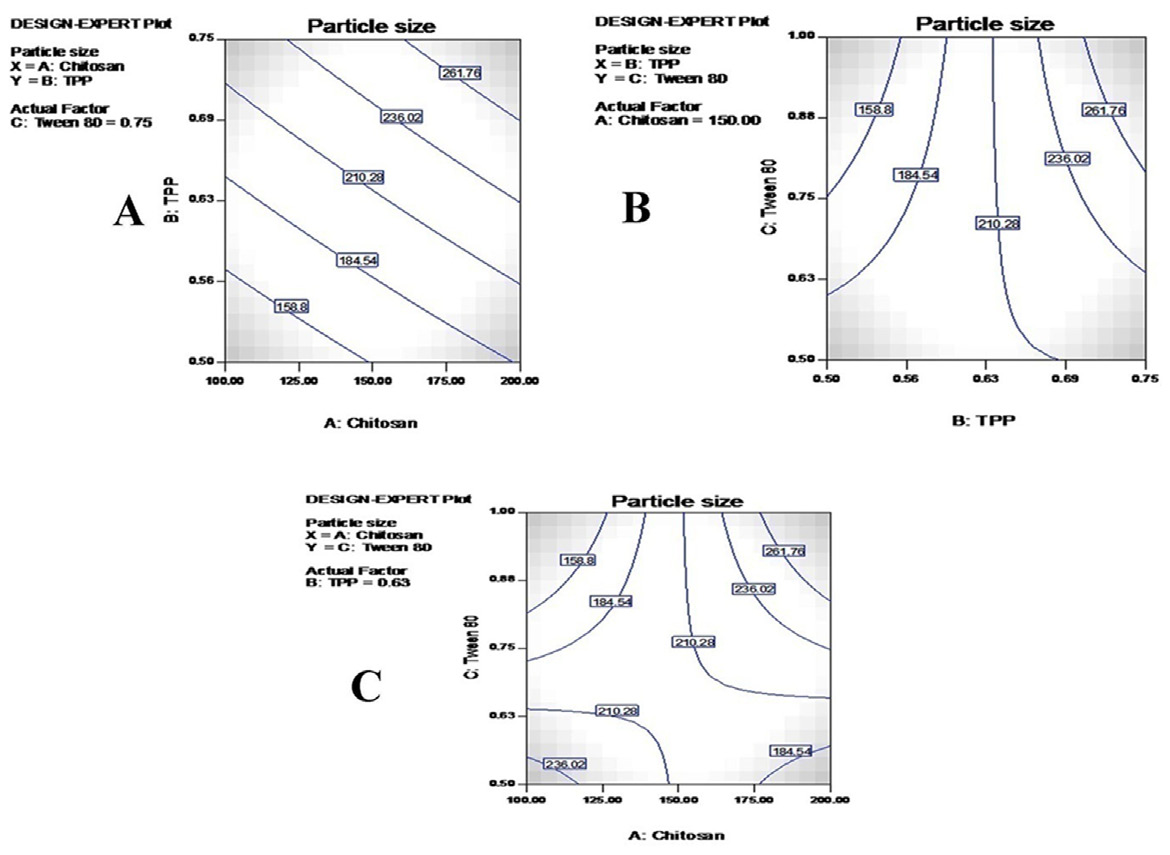
Contour plots for particulate size: The effects of CS and TPP **(A)**, TPP and Tween 80 **(B)** and CS and Tween 80 **(C)** on the particle size of nanoparticles.

Where Y2, A, B and C represents PS, CS, TPP and Tween 80 concentrations, respectively. From the model F-value of 243.23 it was clear that the model is statistically significant. A positive and negative value in the above equation represents synergistic and antagonistic effects of the independent variables, respectively [24]. The correlation coefficient (R^2^) of Y2 was 0.9730, clearly suggesting the 2FI model, as shown in Table 3. Also, a statistically significant effect of the model could be confirmed from the p value for the Y2 model (p < 0.001). All independent variables were found to be statistically significant (A; p<0.0011, B;p=0.0022 and C;p=0.0010), as shown in Table 4. Contour plots are shown in Figure 6.

The formation of nanoparticles takes place due to the interactions between CS, TPP and Tween 80 at a specific concentration range. A wide variation in particle size was found, ranging from 92.50 nm to 482.50 nm. From the results, it was clear that particle size was strongly dependent on the independent variables selected. A direct correlation was observed between CS concentration and the size of the nanoparticles. An increase in particle size was due to the presence of greater amounts of CS in the same volume of acetic acid [30]. Nanoparticle size decreased while TPP concentration increased due to the hardening effect of the TPP, which led to decreased water absorption.

Zeta potentials of nanoparticulate dispersion were found to be between 28.50 to 53.57 mV. Zeta potentials were increased on incorporation of TMZ into nanoparticles and decreased on increasing the concentration of TPP. The cationic nature of CS played a very important role, interacting with the negative charges present on cell membranes, meaning that the drug could be easily released into the cytoplasm of cells [31]. Greater anticancer activity of drug associated with nanoparticles was due to the greater zeta potentials of the particles and hence the stronger interactions with tumor cells. The enhancement in anticancer activity was also due to electrostatic interactions between positively charged amino groups present on CS molecules and negative charges on tumor cells [32]. The interaction between the amino groups of CS and the phosphoric anions of TPP reduced the toxicity of the primary amines of CS42.

### Cytotoxic effect of CS-TMZ-FA-NP

**Figure 7:**
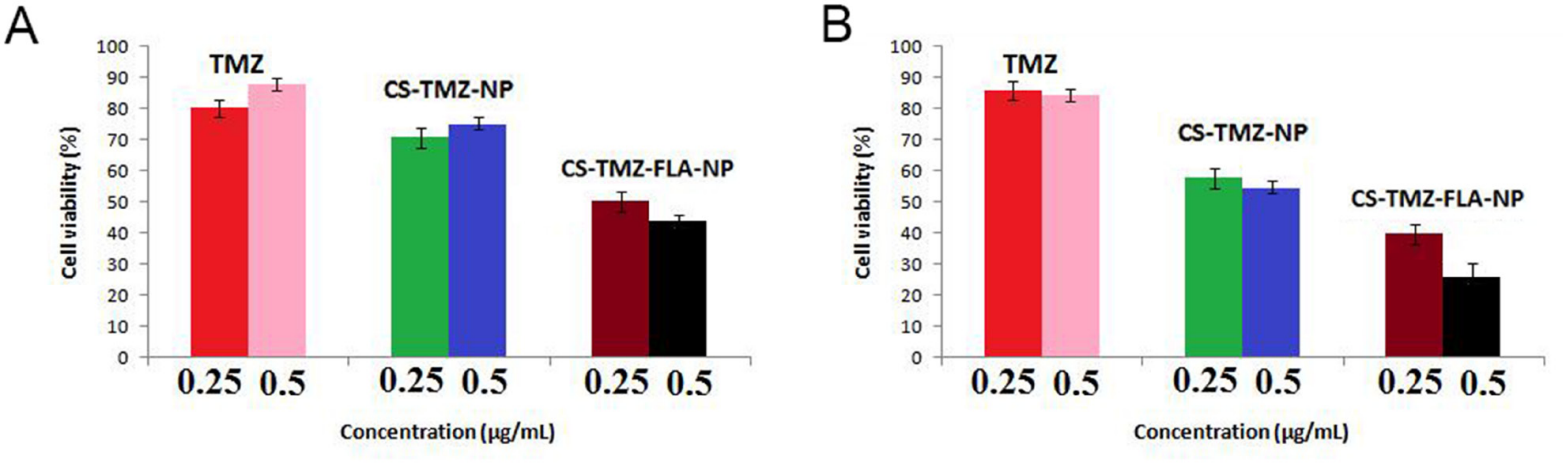
Cell toxicity in human lung cancer cells: Cell toxicity of TMZ, CS-TMZ-NP, and CS-TMZ-FLA-NP in L132 **(A)** and A549 **(B)** cells by MTT assay. Cells were exposed to pure TMZ, CS-TMZ-NP, and CS-TMZ-FA-NP (0.25 and 0.5 μg/ml) for 24 h. The results showed that CS-TMZ-FA-NP produced the greatest antiproliferative effect on these cells compared to TMZ and CS-TMZ-NP.

The cytotoxic assay was performed on a human lung cancer cell line A549 and an epithelial lung cell line L132 *in vitro* using MTT assay. In this assay pure TMZ, CS-TMZ-NP, and CS-TMZ-FA-NP were used to treat the cells for 24 h. As can be seen from the dose-response curve, all test formulations showed a direct relationship between the concentration and cytotoxicity in L132 and A549 cells (Figure 7A and 7B). The antiproliferative effect of CS-TMZ-FA-NP was found to be greater than that of TMZ and CS-TMZ-NP.

### Cellular uptake assays *in vitro*

**Figure 8:**
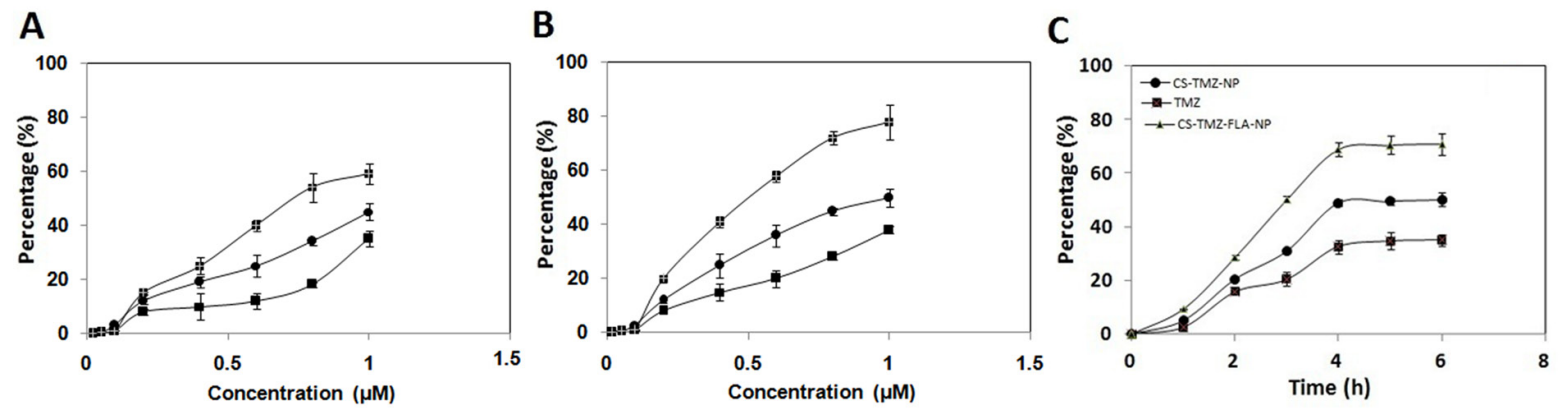
Uptake study on the human lung cancer cells: Evaluation of the dose-dependent uptake of TMZ, CS-TMZ-NP and CS-TMZ-FLA-NP by the L132 cell line **(A)** and A549 cells **(B)**. Cellular uptake of CS-TMZ-FLA-NP and CS-TMZ-NP by both cell lines was time-dependent, but reached saturation after 4 hours of incubation **(C)**. Both cell lines were exposed to TMZ, CS-TMZ-NP, and CS-TMZ-FLA-NP at different concentrations ranging from 0.02 to 1.0 μM for 6 h.

The most important prerequisite for the pharmacological activity of nanoparticles is their ability to get into cancer tissues and cells so that the drug will be available in a sustained and continuous manner. Hence a cellular uptake assay was performed on lung cells. The cells were exposed to TMZ, CS-TMZ-NP and CS-TMZ-FLA-NP (at concentrations of 0.02-1.0 μM) for six hours. Cellular uptake efficiencies of both CS-TMZ-NP and CS-TMZ-FLA-NP in L132 and A549 cells were found to be concentration-dependent (Figure 8A and 8B). However, the uptake efficiency of CS-TMZ-FLA-NP was much higher than that of CS-TMZ-NP (Figure 8A and 8B). Data illustrated in Figure 8C showed that the cellular uptake efficiencies of CS-TMZ-FLA-NP and CS-TMZ-NP in both cell lines were time-dependent, reaching saturation after 4 hours of incubation.

### TMZ pulmonary deposition

**Figure 9:**
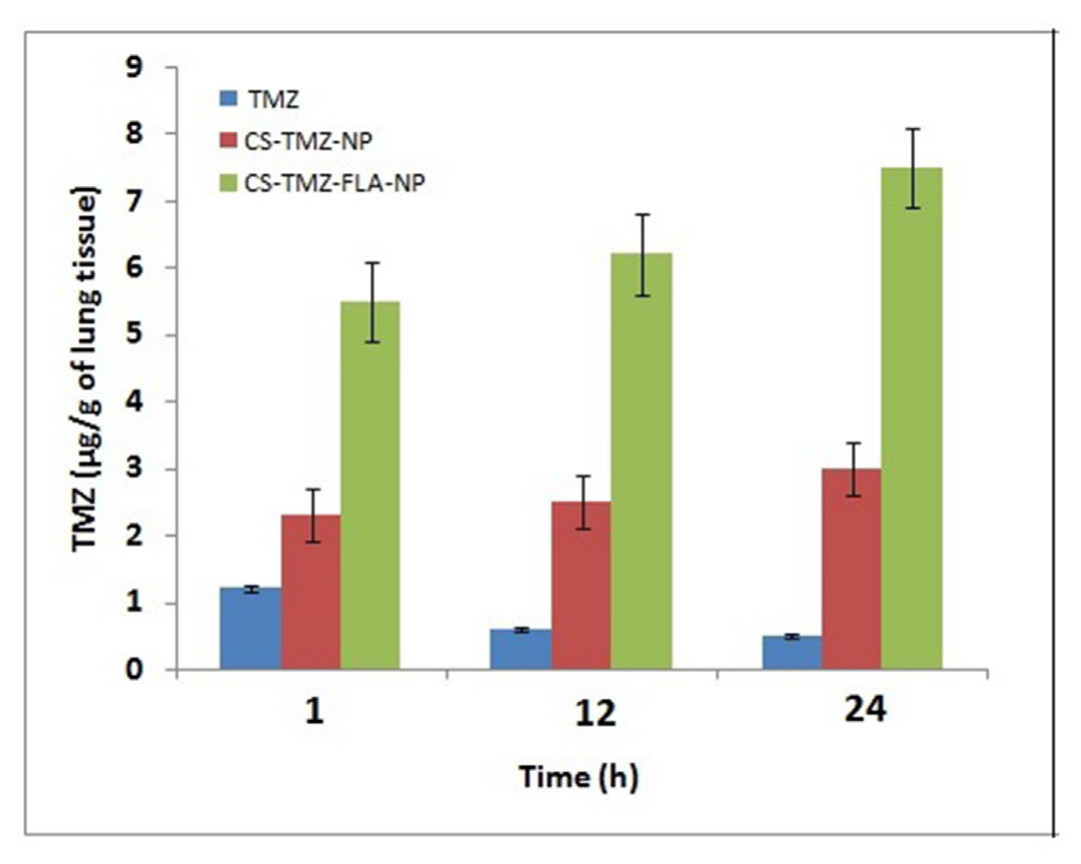
TMZ pulmonary deposition: TMZ concentration deposited in the lungs from free TMZ, CS-TMZ-NP and CS-TMZ-FLA-NP was measured by sacrificing mice at 1, 12, and 24 h post-treatment CS-TMZ-FLA-NP significantly resulted in a higher deposition of TMZ in lung tissues (approximately 7.5 μg/g of lung) compared to TMZ and CS-TMZ-NP. There was continuous improvement in TMZ deposition from CS-TMZ-FLA-NP over the time period indicating that FA surface modification facilitated the accumulation of drug in lung tissues.

For efficient lung cancer therapy, the constant delivery of the active therapeutic moiety from nanoparticles for a prolonged period is necessary. TMZ concentrations deposited in lung tissues from mice treated with TMZ, CS-TMZ-NP, and CS-TMZ-FLA-NP were measured at 1, 12 and 24 h post-treatment. The results showed that the maximum deposition of TMZ in lungs was approximately 1.2 μg/g of lung and this occurred 1 h post-treatment (Figure 9). At 12 h and 24 h post-treatment the concentration of TMZ did not increase further. CS-TMZ-NP produced better TMZ deposition in lung tissue over time. Its maximum deposition of TMZ in lung tissue was approximately 3 μg/g at 24 h post-treatment. In contrast, CS-TMZ-FLA-NP significantly maintained a higher deposition of TMZ in lung tissue (approximately 7.5 μg/g of lung) compared with TMZ and CS-TMZ-NP treatment. There was a continuous improvement in TMZ deposition from CS-TMZ-FLA-NP over the time period studied.

### *In vivo* targeting assay

**Figure 10:**
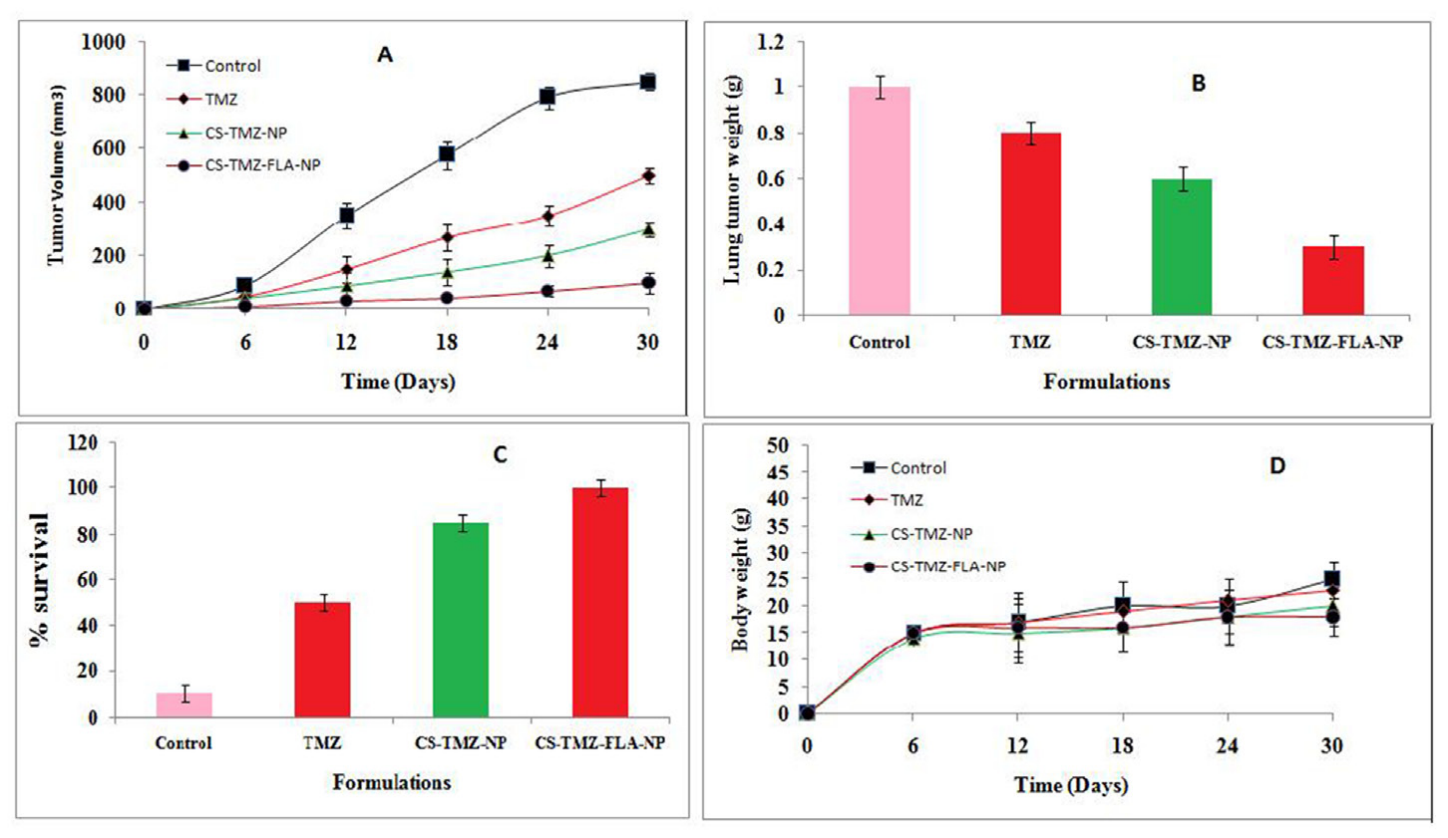
Antitumor efficacy *in vivo*: Antitumor efficacy of TMZ, CS-TMZ-NP and CS-TMZ-FLA-NP in A549 tumor cells in BALB/c-nu/nu athymic mice Tumor volume **(A)**; lung tumor weight **(B)**; percentage survival rate of mice **(C)**; body weight **(D)**.

The antitumor efficacy of TMZ, CS-TMZ-NP, and CS-TMZ-FLA-NP was determined in BALB/c-nu/nu athymic mice bearing A549 tumors *in vivo*. As shown in Figure 10A the tumors in the untreated control group showed an average tumor volume of 850 mm^3^. In contrast, mice treated with formulations and free TMZ exhibited significantly suppressed tumor growth. Out of all the drug treatments, free TMZ resulted in the highest tumor volume of 500 mm^3^. CS-TMZ-NP treatment produced a significant reduction in tumor volume (300 mm^3^), with the maximum antitumor efficacy recorded after CS-TMZ-FLA-NP treatment (tumor volume 100mm^3^).

Throughout the study, a constant tumor volume was maintained by treatment with the CS-TMZ-FLA-NP formulation and a mean of almost 90% tumor regression was observed in this group. This clearly indicates the high efficacy of CS-TMZ-FLA-NP for controlling the spread of cancer metastasis. When tumor volumes were normalized, the CS-TMZ-FLA-NP formulation showed less than a 3 fold increase in the overall tumor volume, which is significantly less than for the control group in which the final tumor volume increased up to 20 times. The efficacy of antitumor activity was found to be in the order of CS-TMZ-FLA-NP >CS-TMZ-NP>TMZ. With respect to the tumor weights (Figure 10B) the CS-TMZ-FLA-NP formulation resulted in the lightest tumors (0.3 g) followed by CS-TMZ-NP (0.6 g). The control group had the largest tumor weights (1.0 g). The maximal antitumor activity of the CS-TMZ-FLA-NP formulation was due to its ability to bind to overexpressed receptors on cell lines, leading to remarkably higher drug concentrations and accumulation within cancerous tissues. The median survival time of animals treated with the CS-TMZ-FLA-NP formulation was found to be 100%. The lowest survival rate at the end of study (only 15%) was observed in the untreated control group (Figure 10C). The safety profile of the formulations could be determined from the changes in body weight of the animals. Figure 10D indicates that no reduction in body weight occurred in any of the groups indicating that the doses of TMZ were well tolerated. The gradual and sustained release of TMZ from nanoparticles contributed to the low toxicity due to prevention of accumulation in normal cells in mice.

### Histopathology and immunohistochemical studies

**Figure 11:**
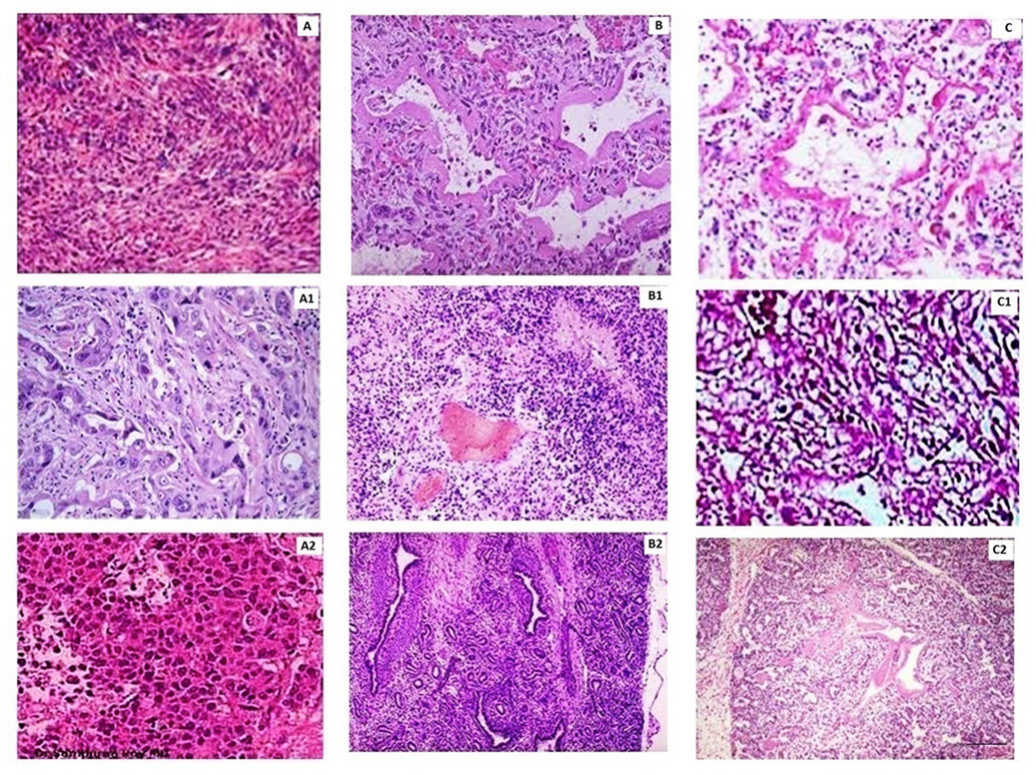
Histopathology and immunohistochemical analysis: **(A)**, histopathology of a section of a tumor from a control group animal; **(B)**, histopathology of a section obtained from a tumor of a mouse treated with CS-TMZ-NP; **(C)**, histopathology of a tumor section obtained from a mouse treated with CS-TMZ-FLA-NP. Immunohistochemical analysis showing cleaved caspase-3 in a tumor section from a control group mouse (A1); a section of a tumor from a mouse treated with CS-TMZ-N (B1) and a section of a tumor from a mouse treated with CS-TMZ-FLA-NP (C1). Immunohistochemical analysis of MMP-9 in a section taken of a tumor collected from a control mouse (A2); a mouse treated with CS-TMZ-N (B2) and a mouse treated with CS-TMZ-FLA-NP (C2). Bar: 50 μm.

It can be seen from Figure 11A, which illustrates the histology of a control tumor that there is a dense and crowded extracellular network in such tissues. However, tumors treated with CS-TMZ-NP (Figure 11B) and with CS-TMZ-FLA-NP (Figure 11C) had extracellular networks missing. Immunohistochemical analysis demonstrated that caspase-3 levels were maximal in groups receiving nanoparticulate formulations compared to the control group (A1<B1<C1), indicating the superior antitumor activity of the formulations. Also, CS-TMZ-NP and CS-TMZ-FLA-NP significantly reduced (A2>B2>C2) the expression of MMP-9 in comparison to the control group indicating potentially excellent results in terms of the treatment of pulmonary tumors. So, it maybe concluded that the elevated levels of caspase-3 and decreased levels of MMP-9 in tumors reinforced the antiproliferative effect of TMZ-loaded formulations. The superior activity of CS-TMZ-FLA-NP in tumors was due to the targeting of the FR, which is overexpressed in lung cancer tumors. Also, the high intracellular deposition and prolonged release of the drug TMZ, which was observed for 45 h, potentially constantly exposes cancer cells to TMZ.

## DISCUSSION

TMZ has been found to be clinically active against lung cancer; however, direct and continuousexposure of tissues to chemotherapeutic agents is associated with undesirable side effects on healthy tissues and cells [33]. Drawbacks of conventional drug delivery systems could be minimized by using a nanotechnological basis for treatments, which can release a chemotherapeutic agent in a sustained manner in a cancerous tissue. Due to the advantages of a nanoparticulate drug delivery system, an attempt has been made here to formulate and optimize targeted nanoparticles for the treatment of lung cancer. In recent years, CS has been explored as a nanoparticulate drug delivery carrier due to its biocompatibility, biodegradability, non-toxicity and its absorption enhancing properties. In addition to this the antioxidant and antitumor activities of CS make it a suitable drug delivery carrier for biomedical applications in cancer treatment [34, 35]. Therefore, in this study we developed CS-TMZ-FLA-NP, which delivers TMZ via targeting FR overexpressed in lung cancer tissues. TMZ is an inactive alkylating agent. It forms the reactive compound, MTIC, at a physiological pH. The cytotoxic effect of MTIC is the result of the alkylation of DNA. This occurs at the O_6_ and N7 positions of guanine and leads to suppression of cell growth. Our data showed that CS-TMZ-FLA-NP could effectively inhibit lung cancer cell growth both *in vitro* and *in vivo*.

Nanoparticles are thought to be initially captured by endocytic cells and then escape to reach acidic lysosomesfrom where the drug is continuously released in a gradual and sustained manner, leading to diffusion into the nuclear complex. Entering cancer tissues and cells so as to deliver drug sustainably and continuously is the most important activity of nanoparticles. The increased antiproliferative effect of CS-TMZ-FA-NP on lung cancer cells might be due to its enhanced cellular uptake. Indeed, comparing the uptake efficiency, we found CS-TMZ-FLA-NP has higher cellular uptake compared with CS-TMZ-NP and TMZ. Moreover, the cellular uptake of CS-TMZ-FLA-NP is concentration-dependent and time-dependent. The maximum uptake of CS-TMZ-FLA-NP might be due to receptor-mediated endocytosis, in which FLA on the CS-TMZ-FLA-NP surface binds to overexpressed receptor FR on cancer cells. The much lower cellular uptake observed with CS-TMZ-NP and TMZ might be due to non-specific adsorption or interaction with cells.

Constant delivery of the active drug from nanoparticles for a prolonged period is critical for effective cancer treatment. In this study, we found that CS-TMZ-FLA-NP had enhanced drug deposition compared with CS-TMZ-NP and TMZ. This improved deposition was most likely to be due to the FLA ligand on the surface of nanoparticles facilitating the accumulation of the drug in lung tissues. Lung cancer cells overexpress FR, which have a strong and specific affinity for the FLA ligand present on nanoparticles. The nanoscale range of the particles easily escapes mucociliary clearance to the lower respiratory tract. Our data suggest that TMZ was released in a controlled and sustained manner helping to maintain a constant exposure of lung tissue to the therapeutic agent. These findings clearly confirm that lung cancer cells that overexpress FR, could be easily recognized by FLA-modified CS nanoparticles and thus result in greater drug accumulation in lung cancer tissue.

## MATERIALS AND METHODS

### Reagents

TMZ was purchased from the Baoji Guokang Bio-Technology Co. (Baoji, Shaanxi, China). CS was obtained from Sigma Aldrich (USA). TPP, acetic acid, folic acid, Tween 80 and N-(3-Dimethyl aminopropyl)-ethylcarbodiimide hydrochloride (EDC) were purchased from Shanghai Chemical Co. (Shanghai, China). Acetonitrile, HPLC-grade water, triethylamine, ortho-phosphoric acid, acetone, anti-caspase-3, anti-MMP-9, and hematoxylin and eosin stains were purchased from Huadong Medical (Hangzhou, China). Miat® nasal insufflator was purchased from MIAT s.p.A. (Milan, Italy). Design expert software (version 6.0.8) was purchased from State-Ease (Minneapolis, USA).

### Animals and cell cultures

BALB/c-nu/nu athymic mice were obtained from the Peking Union Medical College Hospital, China. Cell lines, L132 and A549, were purchased from the Laboratory of Cell and Molecular Biology & State Key Laboratory of Molecular Oncology, Cancer Institute & Cancer Hospital, Chinese Academy of Medical Sciences & Peking Union Medical College (China). Cells were cultured in Dulbecco’s Modified Eagle Medium (supplemented with 10% (v/v) fetal bovine serum, 100 UI/mL penicillin and 100 μg/mL streptomycin sulfate) at at 37°C in a humidified atmosphere with 5% (v/v) CO_2_.

### Three-factor, two-level full factorial experimental design

Three parameters, the concentration of CS, surfactant, and TPP were considered as independent variables. A 3-factor, 2-level full factorial design was used to optimize nanoparticles and to find out the effect of independent variables on the dependent variables, which were percentage encapsulation efficiency and particle size. Independent variables and the different levels are represented in Table 5.

**Table 5:** Variables and the three levels

Independent variable	Low level (−1)	High level (+1)
A= CS Conc. (mg)	100	200
B= TPP Conc. (%)	0.5	0.75
C=Tween 80 Conc. (%)	0.5	1
Dependent Variables		
Y1= EE		
Y2=Particle size		

### Preparation of TMZ-CS nanoparticles by an ionotropic external gelation technique (CS-TMZ-NP)

An ionotropic external gelation method was adopted for the development of CS-TMZ nanoparticles [36]. CS (100 and 200 mg) was dissolved at room temperature in acetic acid (3% v/v, 30 ml) at a stirring speed of 200 rpm in order to obtain a clear solution. Tween-80 (0.5 and 1% v/v) was used as a surfactant in CS solution. TMZ (50 mg) dissolved in organic solution (20 ml acetone) was added dropwise to the aqueous phase of the CS solution. TPP solution was prepared in distilled water (15 ml of 0.5 and 0.75%, w/v). TPP, as the cross-linking agent, was added dropwise at different concentrations into an o/w (oil in water) emulsion with stirring. It was kept overnight for complete evaporation of the organic solvent. A nanoparticulate suspension was formed under the experimental conditions described above. The formation of nanoparticles was associated with the interaction between negative groups from TPP and positive charges from CS. The separation of nanoparticles was carried out by centrifugation (20,000 rpm, 15 min, 10°C). The supernatant obtained after centrifugation was used for determination of non-encapsulated TMZ by HPLC (Young lin, Germany). Supernatant solution (20 μl) was injected into a chromatograph equipped with a UV detector and a C18 column. The mobile phase was acetonitrile/HPLC grade water (85%:15%, v/v) containing 0.16% (w/v) triethylamine and 0.16% (w/v) ortho-phosphoric acid (flow rate 1.5 ml/min; wavelength 240nm). Prepared TMZ-loaded CS nanoparticles (characterized and optimized nanoparticles) were used for the preparation of folic acid-conjugated TMZ-loaded CS nanoparticles.

### Preparation of folic acid-conjugated TMZ-loaded CS nanoparticles (CS-TMZ-FLA-NP)

FLA conjugation was performed on an optimized formulation of CS-TMZ-NP (F4). FLA (5 mg) and EDC (25 mg) were dissolved in 10 ml double distilled water to produce an aqueous solution. EDC was added to the FLA solution to activate the carboxyl groups present on the surface of FLA. This solution was stirred for 1 h for complete activation. This FLA-activated solution was dropped into 10 ml of aphosphate buffer suspension (pH 7.4) containing CS-TMZ-NP (optimized formulation, F4) equivalent to 20 mg of TMZ with magnetic stirring for 15 h in the dark [37]. The separation of nanoparticles was carried out by centrifugation (20,000 rpm, 15 min, 10°C). The supernatant was used for the determination of free FLA by HPLC (Young lin, Germany). Supernatant solution (20 μl) was injected into an HPLC system with a UV detector and C18 column. The mobile phase was acetonitrile/phosphate buffer pH 6.8 (70:30, v/v) containing 0.1% (w/v) triethylamine.

### Freeze drying of folic acid-conjugated TMZ-loaded CS nanoparticles (CS-TMZ-FLA-NP)

The nanoparticulate suspension (CS-TMZ-FLA-NP) containing a cryoprotectant (mannitol 0.2% w/v) and controls were freeze dried using a table-top vacuum freeze dryer (LGJ-18, Sihuan, China). The freeze drying process consisted of two main steps: freezing and vacuum drying.

### Freezing of the nanoparticulate suspension

Glass sample bottles were filled with the nanoparticulate suspension containing cryoprotectant and placed in the cooling trap assembly of the instrument for 7 h at −60°C. During freezing, the temperature was varied from 15°C to −40°C.

### Freeze drying

Sample bottles containing the frozen nanoparticulate suspension were placed in the drying chamber. The chamber was evacuated and the pressure was maintained under 100 Pa. The entire drying cycle was conducted for 28 h with the temperature varying between −30°C and 45°C. The temperature was maintained for 1 h at −30°C and for 1h at −25°C with decreasing temperature up to −5°C. Similarly, for 2 h each the temperature was increased from 0°C to 40°C and at last 4 h at 40°C and 45°C.

### Particle size and morphology

The freeze-dried nanoparticles were suspended in double distilled water and sonicated for 1 min before analysis. Particle size, particle size distribution, and zeta potentials were measured using a Zetasizer (DTS Ver 4.10, Malvern instruments, Germany) at room temperature. Shape and surface morphology were examined by scanning electron microscopy (Leo 435 VP, Cambridge, UK). For zeta potential determination the working distance was maintained at 8.6–8.8 mm with an accelerating voltage of 15.0 kV. The nanoparticles were made electrically conductive by coating them with gold. The gold-coated nanoparticles were mounted on a brass tub using double-sided adhesive tape. During the entire procedure, a vacuum (5 pa) in an ion sputter (Hitachi E1010, Japan) was maintained.

### Drug entrapment efficiency (EE)

The supernatant obtained after centrifugation was used for determining non-encapsulated TMZ by HPLC (Young lin, Germany). Supernatant (20 μl) was injected into a chromatograph equipped with a UV detector and C18 column. The mobile phase was acetonitrile/HPLC grade water (85%:15%, v/v, with0.16% (w/v) trimethylamine and 0.16% (w/v) ortho-phosphoric acid; flow rate 1.5 ml/min, wavelength 240nm. The encapsulation efficiency (E.E) of the nanoparticles was calculated according to the following equation [38]:

E.E (%) = (Total drug-free drug/Total drug) × 100

### Drug loading

Acetone (10 ml) was mixed with CS nanoparticles (10 mg of the TMZ equivalent) dissolved in 50 ml acetic acid (3%, v/v) for proper extraction of drug. The solution was stirred for 3 h for complete extraction of TMZ in acetone. The solution was filtered. TMZ in the filtrate was analyzed by HPLC. The following formula was used to calculate DL: DL(%) = (Amount of drug entrapped/Weight of nanoparticles) x 100.

### Drug release study *in vitro*

A dialysis bag/membrane (molecular cut-off of 5 kDa) method was used to determine TMZ release from nanoparticles. The equivalent of 20 mg TMZ nanoparticles was dispersed in 5 ml phosphate buffer, pH 7.4, in a dialysis tube. The tube was placed in 200 ml PBS solution in a beaker. The temperature of the system was maintained at 37°C and stirred by means of a magnetic stirrer. At a predetermined time interval, 5 ml solution was removed and replenished with fresh solution so as to maintain sink conditions [39]. TMZ release at each time interval was measured by the HPLC method described above.

### Cell culture and cytotoxic assay

The cytotoxic assay was performed in L132 and A549 cells. Cells were seeded for 48 h in 48-well plates (2 × 10^4^ cells/well). Cells were treated with TMZ, CS-TMZ-NP, and CS-TMZ-FLA-NP (0.25 and 0.50 mg/ml), respectively, for 24 h. The MTT assay was used to assess cell viability. At the indicated time point, MTT (1 mg/mL) was added and cells were incubated for another 1 h at 37°C. The formed formazan was dissolved in a 1 M HCl/isopropyl alcohol mixture (1:24, v/v) with shaking for 20 min at room temperature [40].

### Cellular uptake assay *in vitro*

Accumulation of TMZ in cells was determined using a cellular uptake assay. The HPLC method was used for quantification of the cellular uptake of TMZ. Cells (10^4^ cells/well) were seeded in 12-well plates and cultured for 20 h. Cells were treated with test compounds (TMZ, CS-TMZ-NP and CS-TMZ-FLA-NP) at concentrations ranging from 0.02 to 1.0 μM for 6 h. After treatment, cells were lyzed in lysis buffer by sonication. The accumulated TMZ was determined (in supernatants) using HPLC method [41].

### Targeting assay and pulmonary deposition of Temozolomide *in vivo*

A targeting assay was carried out *in vivo* using BALB/c-nu/nu athymic mice. All animals were BALB/c-nu/nu athymic mice with lung cancer xenografts developed as described as follows. A total of 32 animals were divided into 4 groups (n=8). Mice were anesthetized with isoflurane and ∼1.4 × 10^6^ A549 cells were injected at a depth of 4 mm into the left lung. Miat® nasal insufflators were used for administration of formulations by inhalation. TMZ, CS-TMZ-NP, and CS-TMZ-FLA-NP were administered 3 times every 3 days to each mouse at a dose of 4 mg/kg. Antitumor activity was calculated from the tumor volume and tumor weight after sacrificing the mice and lung tumors was isolated. TMZ deposition following TMZ, CS-TMZ-NP, and CS-TMZ-FLA-NP treatments was measured by sacrificing mice at 1, 12, and 24 h. Lungs were isolated and washed with PBS, followed by soaking in 70% (v/v) nitric acid for 12–15 h. The isolated lung tumors were digested at 90°C for 2 h followed by homogenization and centrifugation. The supernatants were collected and analyzed using the HPLC method [42]. All animal experiments were approved by the Laboratory of Cell and Molecular Biology and Institutional Animal Ethics Committee of Peking Union Medical College Hospital, China (Approval number: PUMCH/1045/AEC).

### Histopathological and immunohistochemical studies of tumors

Histopathological analysis was performed using hematoxylin and eosin stain. Sections (20 μm thick) of tumors were obtained and deparaffinised and rehydrated with hematoxylin and eosin stain. For immunohistochemical analysis, tumor samples were stored in formalin solution under frozen conditions, rehydrated using alcohol, and embedded in paraffin. Thin paraffin sections (15 μm) were cut and mounted on slides coated with poly-L-lysine. Deparaffinised sections were rehydrated using alcohol and 3% (v/v) hydrogen peroxide. Sections were blocked with a protein blocking solution for 1 h, followed by incubation with caspase-3 and MMP-9 antibodies and exposure to chromogen for color development.

## CONCLUSION

We have successfully developed the CS-TMZ-FLA-NP by an inotropic gelation method in order to target FR, which are overexpressed in lung cancer tissues. CS-TMZ-FLA-NP showed the greatest antiproliferative efficacy in the lung cancer cells when compared to free TMZ and CS-TMZ-NP. Moreover, we found that CS-TMZ-FLA-NP maintained a higher deposition of TMZ in lung tumor tissues. Mice treated with CS-TMZ-FLA-NP had a 100% survival rate with an accompanying significant suppression of tumor growth. Furthermore, histopathological and immunohistochemical studies demonstrated a superior anticancer activity of CS-TMZ-FLA-NP with respect to pulmonary tumors in mice. The results presented indicate that CS-TMZ-FLA-NP can effectively facilitate the targeting of TMZ to lung cancer cell lines and tumors in a sustained manner thereby improving the therapeutic efficacy of TMZ. Such a targeted drug delivery system holds great potential for the development of much needed and improved lung cancer treatments.

## Abbreviations

TMZ: temozolomide
CS: chitosan
CS-TMZ-NP: temozolomide loaded chitosan nanoparticles
CS-TMZ-FLA-NP: folic acid conjugated temozolomide loaded chitosan nanoparticles
FLA: folic acid
FR: folic acid receptors
SCLC: small cell lung carcinoma
NSCLC: non small cell lung carcinoma
MTIC: 5-(3-methyltriazen-1-yl) imidazole-4-carboxamide
TPP: tripoly phosphate

## References

[1] Grenha A, Seijo B, Remuñán-López C (2005). Microencapsulated chitosan nanoparticles for lung protein delivery. Eur J Pharm Sci.

[2] Califano R, Abidin AZ, Peck R, Faivre-Finn C, Lorigan P (2012). Management of small cell lung cancer: recent developments for optimal care. Drugs.

[3] Jemal A, Bray F, Center MM, Ferlay J, Ward E, Forman D (2008). Global cancer statistics. CA Cancer J Clin.

[4] Koshkina NV, Waldrep JC, Roberts LE, Golunski E, Melton S, Knight V (2001). Paclitaxel liposome aerosol treatment induces inhibition of pulmonary metastases in murine renal carcinoma model. Clin Cancer Res.

[5] Zarogoulidis P, Chatzaki E, Porpodis K, Domvri K, Hohenforst-Schmidt W, Goldberg EP, Karamanos N, Zarogoulidis K (2012). Inhaled chemotherapy in lung cancer: future concept of nanomedicine. Int J Nanomedicine.

[6] Okamoto H, Shiraki K, Yasuda R, Danjo K, Watanabe Y (2011). Chitosan-interferon-β gene complex powder for inhalation treatment of lung metastasis in mice. J Control Release.

[7] Lefranc F, Facchini V, Kiss R (2007). Proautophagic drugs: a novel means to combat apoptosis-resistant cancers, with a special emphasis on glioblastomas. Oncologist.

[8] Trinh VA, Pastel SP, Hwu WJ (2009). The safety of temozolomide in the treatment of malignancies. Expert Opin Drug Saf.

[9] Maldonado F, Limper AH, Lim KG, Aubry MC (2007). Temozolomide-associated organizing pneumonitis. Mayo Clin Proc.

[10] Adonizio CS, Babb JS, Maiale C, Huang C, Donahue J, Millenson MM, Hosford M, Somer R, Treat J, Sherman E, Langer CJ (2002). Temozolomide in non-small-cell lung cancer: preliminary results of a phase II trial in previously treated patients. Clin Lung Cancer.

[11] Jain A, Jain SK (2013). Formulation and optimization of temozolomide nanoparticles by 3factor 2 level factorial design. Biomatter.

[12] Hegi ME, Diserens AC, Gorlia T, Hamou MF, Tribolet N, Weller M, Kros JM, Hainfellner JA, Mason W, Mariani L, Bromberg JE, Hau P, Mirimanoff RO (2005). MGMT gene silencing and benefit from temozolomide in glioblastoma. N Engl J Med.

[13] Pohlmann AR, Weiss V, Mertins O, Silveira NP, Guterres SS (2002). Spray-dried indomethacin loaded polyester nanocapsules and nanospheres: development, stability evaluation and nanostructure models. Eur J Pharm Sci.

[14] Huang M, Ma Z, Khor E, Lim LY (2002). Uptake of FITC-Chitosan nanoparticles by A549 Cells. Pharm Res.

[15] Duret C, Wauthoz N, Sebti T, Vanderbist F, Amighi K (2012). New inhalation-optimized itraconazole nanoparticle-based dry powders for the treatment of invasive pulmonary aspergillosis. Int J Nanomed.

[16] Wu XS, Wang N (2001). Synthesis, characterization, biodegradation, drug delivery application of biodegradable lactic/glycolic acid polymers. Part II: biodegradation. J Biomat Sci Polym Ed.

[17] Sinha VR, Singla AK, Wadhawan S, Kaushik R, Bansal K, Dhawan S (2004). Chitosan microspheres as a potential carrier for drugs. Int J Pharm.

[18] Calvo P, Vila-Jato JL, Alonso MJ (1997). Evaluation of cationic polymer-coated nanocapsules as ocular drug carriers. Int J Pharm.

[19] Senel S, McClure S (2004). Potential applications of chitosan in veterinary medicine. Adv Drug Deliv Rev.

[20] Weitman SD, Lark RH, Coney LR (1992). Distribution of the folate receptor GP38 in normal and malignant cell lines and tissues. Cancer Res.

[21] Zhang Y, Guo L, Roeske RW, Antony AC, Jayaram HN (2004). Pteroyl-γ-glutamate-cysteine synthesis and its application in folate receptor-mediated cancer cell targeting using folate-tethered liposomes. Anal Biochem.

[22] Antony AC (1992). The biological chemistry of folate receptors. Blood.

[23] Dash V, Mishra SK, Singh M, Goyal AK, Rath G (2010). Release kinetic studies of aspirin microcapsules from ethyl cellulose, cellulose acetate phthalate and their mixtures by emulsion solvent evaporation method. Sci Pharm.

[24] Chaudhary H, Gauri S, Rathee P, Kumar V (2013). Development and optimization of fast dissolving oro-dispersible films of granisetron HCl using Box–Behnken statistical design. Bull Fac Pharm. Cairo Univ.

[25] Aspden TJ, Mason JD, Jones NS, Lowe J, Skaugrud O, Illum L (1997). Chitosan as a nasal delivery system: the effect of chitosan solutions on *in vitro* and *in vivo* mucociliary transport rates in human turbinates and volunteers. J Pharm Sci.

[26] Janes KA, Calvo P, Alonso MJ (2001). Polysaccharide colloidal particles as delivery systems for macromolecules. Adv Drug Deliv Rev.

[27] Papadimitriou S, Bikiaris D, Avgoustakis K, Karavas E, Georgarakis M (2008). Chitosan nanoparticles loaded with dorzolamide and pramipexole. Carbohydr Polym.

[28] Ahmed TA, El-Say KM (2014). Development of alginate-reinforced chitosan nanoparticles utilizing W/O nanoemulsification/internal crosslinking technique for transdermal delivery of rabeprazole. Life Sci.

[29] Yang W, Peters JI, Williams RO (2008). Inhaled nanoparticles--a current review. Int J Pharm.

[30] Ahlin P, Kristl J, Kristl A, Vrecer F (2002). Investigation of polymeric nanoparticles as carriers of enalaprilat for oral administration. Int J Pharm.

[31] Bozkir A, Saka OM (2004). Chitosan nanoparticles for plasmid DNA delivery effect of chitosan molecular structure on formulation and release characteristics. Drug Deliv.

[32] Choksakulnimitr S, Masuda S, Tokuda H, Takakura Y, Hashida M (1995). *In vitro* cytotoxicity of macromolecules in different cell culture systems. J Control Release.

[33] Mehrotra A, Nagarwal RC, Pandit JK (2011). Lomustine loaded chitosan nanoparticles: characterization and *in-vitro* cytotoxicity on human lung cancer cell line L132. Chem Pharm Bull (Tokyo).

[34] Pandey R, Khuller GK (2006). Oral nanoparticle-based antituberculosis drug delivery to the brain in an experimental model. J Antimicrob Chemother.

[35] Illum L (1998). Chitosan and its use as a pharmaceutical excipient. Pharm Res.

[36] Du WL, Niu SS, Xu YL, Xu ZR, Fan CL (2009). Antibacterial activity of chitosan tripolyphosphate nanoparticles loaded with various metal ions. Carbohydr Polym.

[37] Song H, Su C, Cui W, Zhu B, Liu L, Chen Z, Zhao L (2013). Folic acid-chitosan conjugated nanoparticles for improving tumor-targeted drug delivery. Biomed Res Int.

[38] Allemann E, Gurny R, Deolker E (1993). Drug-loaded nanoparticles—preparation methods and drug targeting issues. Eur J Pharm Biopharm.

[39] Calvo P, Remuñán-López C, Vila-Jato JL, Alonso MJ (1997). Novel hydrophilic chitosan-polyethylene oxide nanoparticles as protein carriers. J Appl Polym Sci.

[40] Singh P, Gupta U, Asthana A, Jain NK (2008). Folate and folate-PEG-PAMAM dendrimers: synthesis, characterization, and targeted anticancer drug delivery potential in tumor bearing mice. Bioconjug Chem.

[41] Liu J, Liu J, Chu L, Wang Y, Duan Y, Feng L, Yang C, Wang L, Kong D (2011). Novel peptide–dendrimer conjugates as drug carriers for targeting nonsmall cell lung cancer. Int J Nanomedicine.

[42] Long JT, Cheang TY, Zhuo SY, Zeng RF, Dai QS, Li HP, Fang S (2014). Anticancer drug-loaded multifunctional nanoparticles to enhance the chemotherapeutic efficacy in lung cancer metastasis. J Nanobiotechnology.

